# WNT and inflammatory signaling distinguish human Fallopian tube epithelial cell populations

**DOI:** 10.1038/s41598-020-66556-y

**Published:** 2020-06-17

**Authors:** Ian M. Rose, Mallikarjun Bidarimath, Alex Webster, Andrew K. Godwin, Andrea Flesken-Nikitin, Alexander Yu. Nikitin

**Affiliations:** 1 Department of Biomedical Sciences and Cornell Stem Cell Program, Cornell University, Ithaca, New York USA; 2 University of Kansas Cancer Center, Kansas City, Kansas USA; 3 Department of Pathology & Laboratory Medicine, University of Kansas Medical Center, Kansas City, Kansas USA

**Keywords:** Ovarian cancer, Data processing, Adult stem cells

## Abstract

Many high-grade serous carcinomas (HGSCs) likely originate in the distal region of the Fallopian tube’s epithelium (TE) before metastasizing to the ovary. Unfortunately, molecular mechanisms promoting malignancy in the distal TE are obfuscated, largely due to limited primary human TE gene expression data. Here we report an in depth bioinformatic characterization of 34 primary TE mRNA-seq samples. These samples were prepared from proximal and distal TE regions of 12 normal Fallopian tubes. Samples were segregated based on their aldehyde dehydrogenase (ALDH) activity. Distal cells form organoids with higher frequency and larger size during serial organoid formation assays when compared to proximal cells. Consistent with enrichment for stem/progenitor cells, ALDH+ cells have greater WNT signaling. Comparative evaluation of proximal and distal TE cell population’s shows heightened inflammatory signaling in distal differentiated (ALDH−) TE. Furthermore, comparisons of proximal and distal TE cell populations finds that the distal ALDH+ TE cells exhibit pronounced expression of gene sets characteristic of HGSC sub-types. Overall, our study indicates increased organoid forming capacity, WNT/inflammatory signaling, and HGSC signatures underlie differences between distal and proximal regions of the human TE. These findings provide the basis for further mechanistic studies of distal TE susceptibility to the malignant transformation.

## Introduction

Ovarian/extra-uterine high-grade serous carcinoma (HGSC) is the most common and most lethal gynecological malignancy, accounting for nearly 14,000 deaths per year in the United States^1^. There is mounting evidence indicating that serous tubal intraepithelial carcinomas (STICs) are precursor lesions to some HGSCs^2–4^. Moreover, *in vivo* perturbations of TP53, MYC, and hTERT and RB family genes, which are associated with pathways frequently perturbed in HGSC^1^, cause human TE cells to adopt traits reminiscent of STICs/HGSCs^5^. It has been noted that STICs tend to occur more frequently in the distal region (closer to the ovary) than in the proximal region (farther from the ovary) of the Fallopian tube, also known as the uterine tube^3,6,7^.

Stem cells are frequently implicated in malignant transformation^7,8^, thus regional differences in the TE stem cells may account for the distal TE’s tendency to harbor STICs. Indeed, previous human and mouse studies based on immunohistochemical and long-term labeling analysis have suggested that stem/progenitor cells may occur more frequently in the distal TE^9–12^. Support for this notion also comes from the observations of preferential sphere formation by human distal TE^9^ and organoid formation of the mouse distal oviduct (the mouse analogue to the Fallopian tube^13^). Long-term organoid formation assays have been indicative of adult tissue stem cells being present in human TE cells isolated from both the proximal and distal regions of the Fallopian tube^14^. However, quantitative comparisons of proximal and distal TE organoid capacity have not been performed. Additionally, studies which interrogate organoid-forming cells, performing quantitative organoid assays and measure global gene expression data in primary human TE are sparse or absent.

Differences between TE stem cell populations are not the only factors that may promote malignant transformation in the distal TE. Chronic inflammation is known to cause cancer in a number of contexts^15^. The ovary is known to release inflammatory factors on a regular basis in humans (^16,17^ and Supplementary Figure 1). The expression of pro-inflammatory cytokine IL-8 has been shown to correlate with ovulation^18^. Consequently, the ovary-derived factors have long been suspected of promoting malignant transformation^19^. More recent studies find that follicular fluid induces DNA damage and proliferation in TE^20,21^, and exposure to follicular fluid also induces changes in TE reminiscent of STICs^22^. Gene expression data from different human TE cell populations may aid in determining the immediate relevance of these observations to the human TE.

Given that information pertaining to TE stem and differentiated cells is sparse, and gene expression data in primary human cells is very limited, we devised a fluorescence activated cell sorting (FACS) strategy based on ALDH activity to purify populations of stem/progenitor and differentiated epithelial enriched cell populations from both the proximal and distal regions of the human TE. Aldehyde dehydrogenase (ALDH) is a detoxifying enzyme and its increased activity is frequently observed in stem/progenitor cells of ovarian surface epithelium, mammary, prostate, colon, haematopoietic, neural and mesenchymal cell lineage^8,23,24^. Long term organoid formation assays demonstrate that ALDH+ cell populations have a greater capacity for organoid formation than ALDH− cell populations. Based on a thorough bioinformatic characterization of the isolated cell populations we also report that ALDH+ cells have greater WNT signaling activity and that the distal TE is characterized by increased inflammatory signaling and gene expression patterns reminiscent of HGSC.

## Results

### ALDH activity distinguishes organoid-forming cells

**Figure 1:**
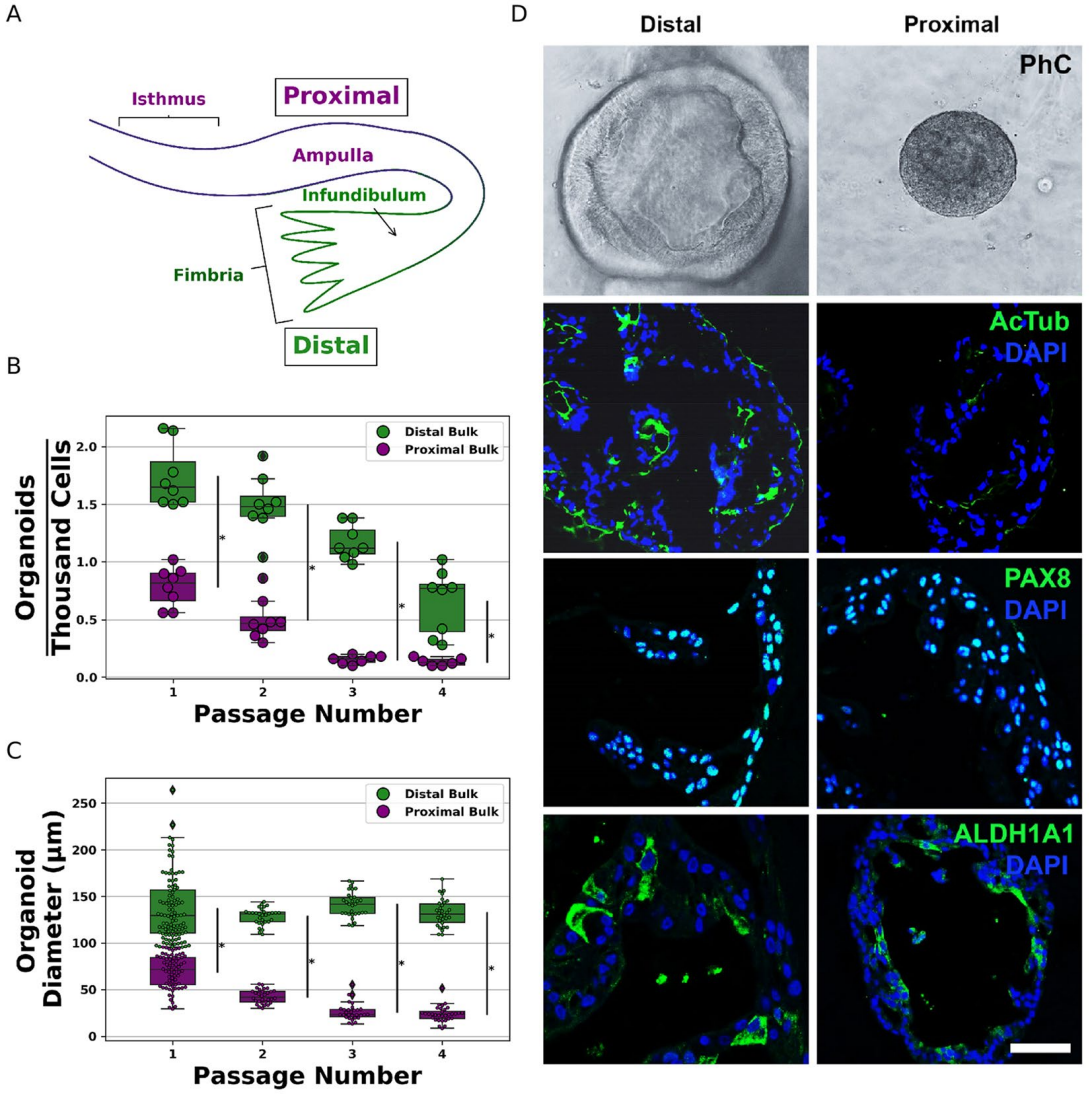
Bulk Fallopian tube organoid formation assays. (**A**) Schematic of the proximal and distal regions of Fallopian tubes. The frequency (**B**) and the diameter (**C**) of the distal and proximal TE orgnoids were measured on the 8th day in the culture successively for 4 passages. Each dot corresponds to an observation. (**D**) Representative images of human distal and proximal tube epithelial organoids. First row shows the phase contrast (PhC) images of the organoids. The expression of ACTUB (second row), PAX8 (third row), and ALDH1A1 (fourth row) in distal and proximal tubal epithelial organoids. Note the cilia are marked by ACTUB (green), nuclear staining (green) for PAX8 and cytoplasmic staining (green) for ALDH1A1. Counterstaining with DAPI for immunofluorescence staining. Bar, D, First row, 150 μm; Second row, 60 μm; third row, 50 μm; and fourth row, 50 μm.

All Fallopian tubes used in our experiments were removed from donors not afflicted with ovarian cancer and not carrying mutant BRCA1/2 alleles, and who were between the ages of 32 and 51. Proximal and distal regions of the TE were divided as indicated in Fig. 1A. To test if there are regional differences in TE organoid formation, we prepared organoids from proximal and distal Fallopian tube regions and propagated them for 4 passages. Consistent with previous report^14^, primary TE cells from both distal and proximal regions were able to form organoids (Fig. 1B). However, distal TE cells consistently formed organoids at a significantly higher frequency than their proximal region counterparts (Fig. 1C). Furthermore, organoids grown from the distal TE region tend to be significantly larger than their proximal counterparts (Fig. 1D). Both distal and proximal organoids contained ciliated (AcTub+) and secretory (PAX8+) cells, as well as cells expressing stem/progenitor cell marker ALDH1A1.

**Figure 2:**
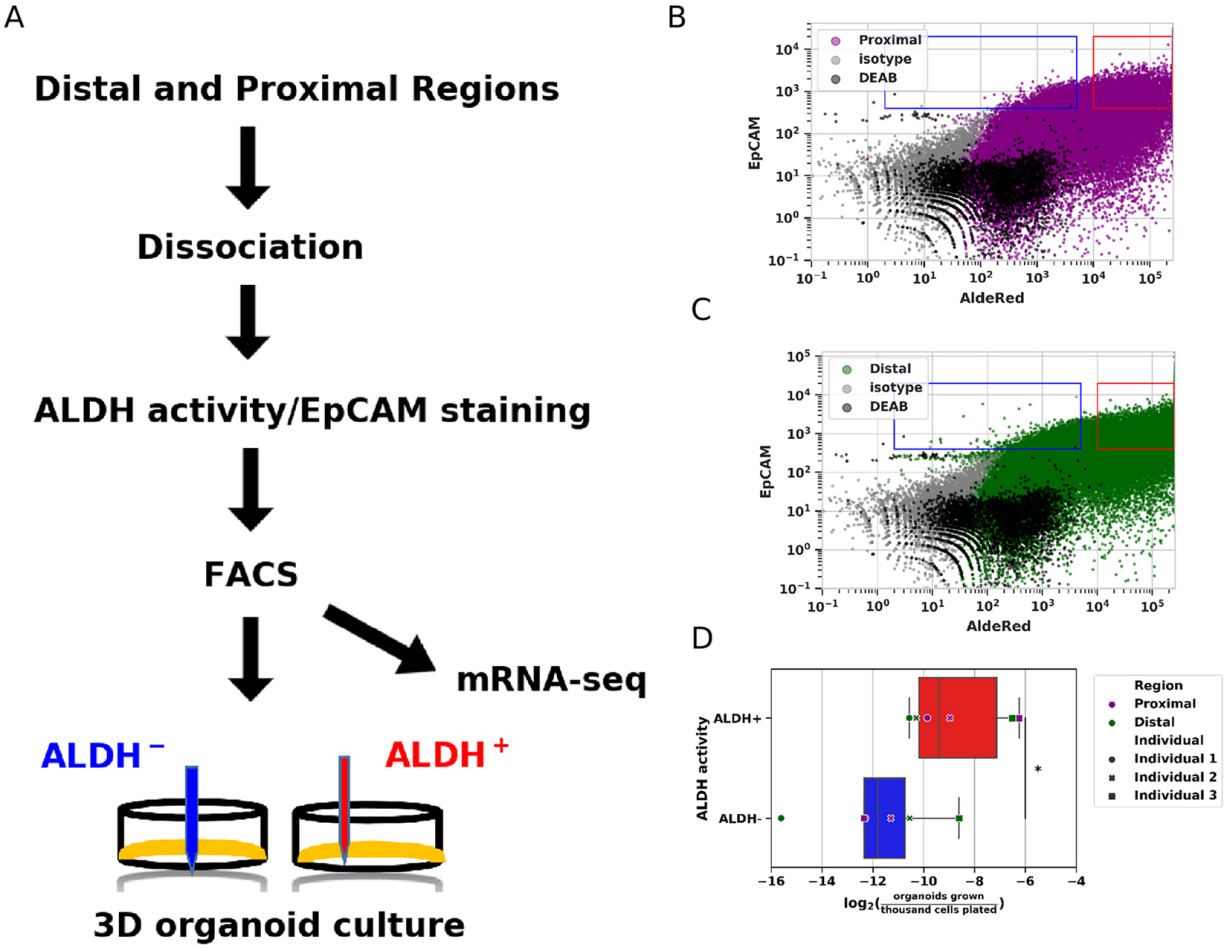
Organoid assays from Fallopian tube epithelial cells isolated by ALDH activity. (**A**) Schematic describing experimental strategy. Epithelial cells from cryopreserved proximal and distal region Fallopian tube fragments were isolated by FACS on the basis of ALDH activity. (**B-C**) Representative FACS data. (**B**) Gates for proximal EpCAM+/ALDH− cells (blue rectangle) and EpCAM+/ALDH+ cells (red rectangle). Grey and black dots indicate EpCAM isotype control cells and cells treated with an ALDH inhibitor (DEAB), respectively. (**C**) Gates for distal EpCAM+/ALDH− cells (blue rectangle) and EpCAM+/ALDH+ cells (red rectangle). Grey and black dots indicate EpCAM isotype control cells and cells treated with an ALDH inhibitor (DEAB), respectively. (**D**) Organoid formation by EpCAM+ ALDH+ and EpCAM+ ALDH− cells isolated from proximal (purple markers) and distal (dark green markers) regions of Fallopian tubes.

Based on previous observations that ALDH activity is frequently observed in stem/progenitor cells, we hypothesized that ALDH+ epithelial (EpCAM+) cell populations have increased organoid formation as compared to ALDH−/EpCAM+ cells. Therefore, we FACS isolated viable EpCAM+/ALDH+ and EpCAM+/ALDH− cell populations from the proximal and distal regions of Fallopian tubes. After determining that sample storage time and Fallopian tube region do not seem to significantly affect each sample’s epithelial cell composition (Supplementary Figure 2), we conducted organoid formation assays as diagramed in Fig. 2A. Light scatter gating was used to exclude debris (Supplementary Figure 3, Supplementary Table 1) before collecting viable (Supplementary Figure 3) EpCAM+/ALDH+ and EpCAM+/ALDH− cells from proximal (Fig. 2B) and distal (Fig. 2C) regions of the TE. We have found that both proximal and distal TE EpCAM+/ALDH+ and EpCAM+/ALDH− cell populations have the capacity to form organoids and that significantly more organoids have formed from EpCAM+/ALDH+ isolates, as compared to EpCAM+/ALDH− cell population, in both proximal and distal samples (Fig. 2D, Supplementary Table 2). Organoid formation is generally indicative of stem/progenitor cells *ex vivo*. Thus, these findings suggest that ALDH activity is a suitable means of enriching for stem/progenitor cells in human TE isolates.

### Proximal and distal TE cell populations display distinct gene expression patterns

Having determined that ALDH activity is a suitable criterion for enriching for stem/progenitor cells, we created mRNA-seq libraries for 34 samples (7 proximal EpCAM+/ALDH−, 9 proximal EpCAM+/ALDH+, 10 distal EpCAM+/ALDH−, 8 distal EpCAM+/ALDH+, Supplementary Table 3) from 12 generally healthy donors. Following data pre-processing (see Methods) we applied NGS checkmate^25^ to verify that each library originated with the individual indicated by our records (Supplementary Figure 4). To validate our FACS strategy and identify any contamination present in our mRNA-seq samples, we performed a deconvolution analysis using the R package BSEQsc^26^ with recently published distal Fallopian tube single-cell mRNA-seq data^27^. We found that contamination from non-epithelial cells was minimal (Supplementary Figure 5). Even so, we tested whether the extent of T-cell or smooth muscle cell (the two contaminating cell types detected) contamination explained a statistically significant amount of variation in the expression of any genes. We found a significant affect in only 4 genes (Supplementary Figure 6). Therefore, we conclude it is unlikely that contamination by non-epithelial cell types confounds out results. As a final quality check, we performed Gene Set Enrichment Analysis (GSEA)^28^ using expressed ALDH family proteins and found that ALDH gene expression is significantly up-regulated in EpCAM+/ALDH+samples (Supplementary Figure 7).

**Figure 3:**
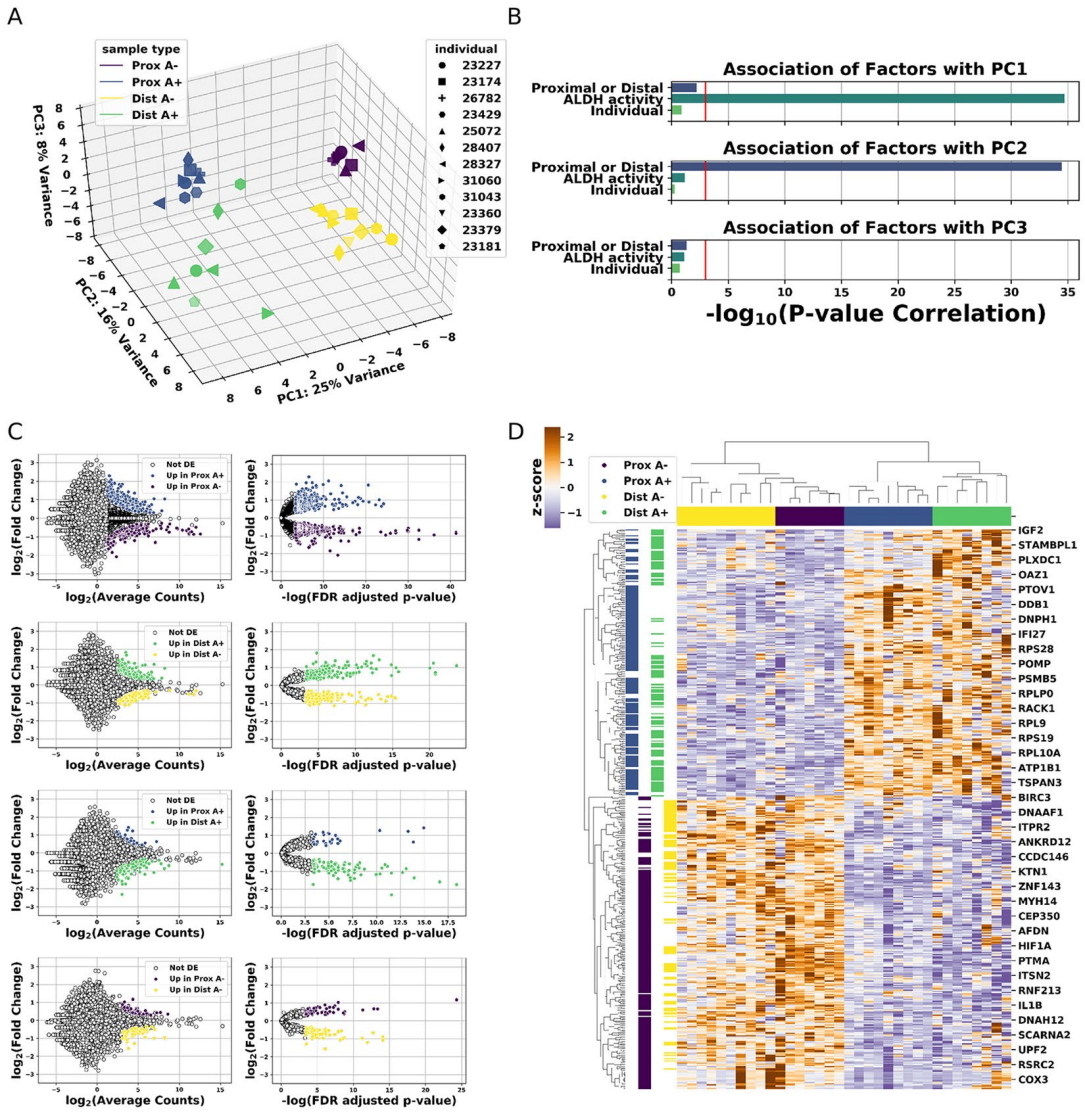
Gene expression data from primary human proximal and distal EpCAM+/ALDH− and EpCAM+/ALDH+ cell populations. (**A**) Principle component (PC) plot of the first three PCs from human TE mRNA-seq samples. Each dot belongs to a population indicated by the dot’s color. The dot’s shape corresponds to the individual that donated the corresponding sample’s Fallopian tube fragment. (**B**) Bar plots indicating the significance of the association between the indicated PC and the covariate listed on the y-axis. The x-axis corresponds to the statistical significance of the association for each factor on the y-axis and the PC indicated by the title of each subplot. The red line corresponds to a p-value of 0.05. Bars that meet or pass to the right of the red line have a p-value less than 0.05. (**C**) Differentially expressed genes in human TE populations. Each MA and volcano plot pair displays genes which are differentially expressed between the cell populations indicated in the MA plot’s legend. (**D**) Heatmap summarizing sample segregation by, and expression of differentially expressed genes. Colors at the top of each column indicate which population the column belongs to. Each row corresponds to a gene found to be differentially expressed in at least one comparison. The colors along each row indicate which population(s) the gene in that row is differentially expressed in.

Principal component (PC) analysis has found the 4 cell populations segregate into visually distinct groups (Fig. 3A). To determine the extent to which variation in the mRNA-seq samples is associated with our experimental design, or potentially confounding factors, we checked the significance of the association of the first 3 PCs with the Fallopian tube region each sample originated in, ALDH activity of that sample, as well as with the individual each sample came from. We chose to examine Fallopian tube region and ALDH activity, as these were the criteria on which we FACS isolated the cells. We chose to examine individual because the individual each sample came from seemed the factor most likely to confound our analysis. Of the potential covariates we tested, ALDH activity and region of origin correlated most strongly with PC1 and PC2. Importantly, the individual that donated sample material was not significantly correlated with any of the first PCs (Fig. 3B).

Having observed the high correlation between ALDH activity and region of sample origin with PC1 and PC2, we performed differential expression analysis using the DESeq2^29^ R package. As expected, stem and differentiated cell enriched populations recover greater numbers of differentially expressed genes than comparisons between proximal and distal populations (Fig. 3C, Supplementary Tables 4–7). An overview of expression differences, which display expression trends distinguishing proximal and distal TE, is given in Fig. 3D.

### Stem cell enriched populations exhibit increased Wnt signaling compared to differentiated cell enriched populations

**Figure 4:**
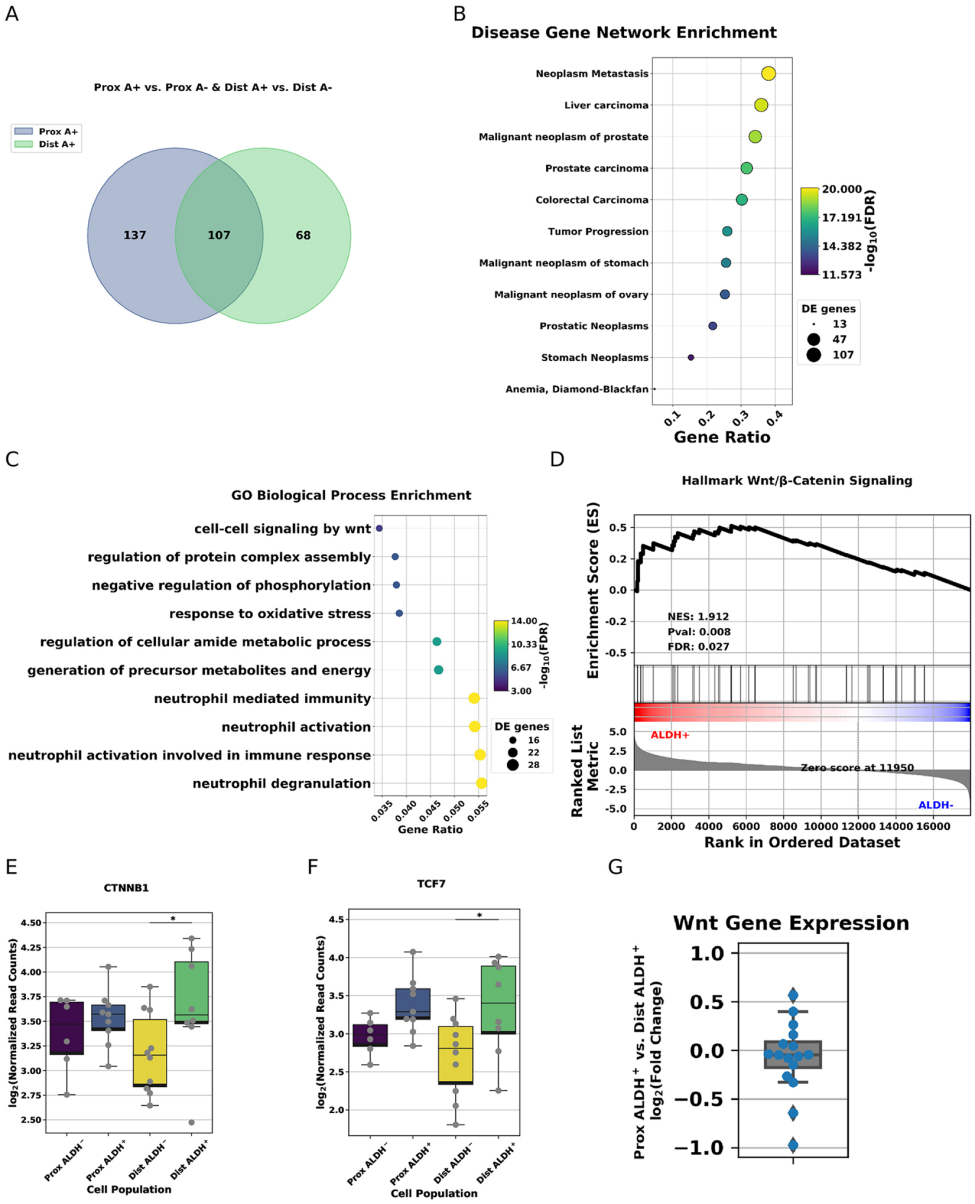
Functional significance of differentially expressed genes. (**A**) Venn diagram summarizing the extent to which differentially expressed genes from comparisons made in Fig. 3C overlap. (**B**) Top 10 Disease Gene Net enrichment results sorted by multiple test corrected p-value for the 312 genes upregulated in proximal and distal EpCAM+/ALDH+ cell populations compared to their EpCAM+/ALDH− counterparts. (**C**) Top 10 GO Biological Process enrichment results sorted by hits/background gene for the same 312 genes as Fig. 4B. (**D**) GSEA results for Hallmark Wnt/β-Catenin signaling. (**E**) Boxplots displaying β-Catenin signaling in each of the four cell populations. (**F**) Boxplots plots displaying TCF7 expression in each of the four cell populations. (**G**) Log_2_(Fold Change) for cell-cell signaling by Wnt genes between proximal and distal EpCAM+/ALDH+ cell populations.

To contextualize our differential expression results, we conducted gene ontology enrichment analysis using genes that are upregulated in EpCAM+/ALDH+ populations compared to their EpCAM+/ALDH− counterparts (Fig. 4A). Stem/progenitor cells can play a role in malignant transformation and so we have begun by searching genes up regulated in EpCAM+/ALDH+ populations for enrichment in Disease Gene Network^[330^. We have found top hits relating to metastatic disease (Fig. 4B, Supplementary Table 8). Querying GO Biological Process also recovered ‘cell-cell signaling by wnt’ as a prominent, statistically significant result (Fig. 4C, Supplementary Table 9). We continued by performing GSEA on EpCAM+/ALDH+ vs. EpCAM+/ALDH− cell enriched populations (See Supplementary Table 10 for all gene sets used in this study). GSEA identified enrichment of the Hallmark Wnt/β-Catenin Signaling gene set (Fig. 4D). β-catenin and TCF family transcription factors are important mediators of canonical WNT signaling, which is an important pathway in maintaining SC self-renewal and cancer. We have found that distal EpCAM+/ALDH+ cell populations also show significant up regulation of *CTNNB1* (often referred to as β-Catenin, Fig. 4E) and *TCF7* (Fig. 4F) compared their EpCAM+/ALDH− counterparts. To see if WNT signaling may distinguish proximal from distal EpCAM+/ALDH+ populations, we examined the expression fold changes between genes annotated as involved in the WNT Signaling GO Biological Process (Fig. 4G).

### Inflammatory signaling is more pronounced in distal TE cell populations

**Figure 5:**
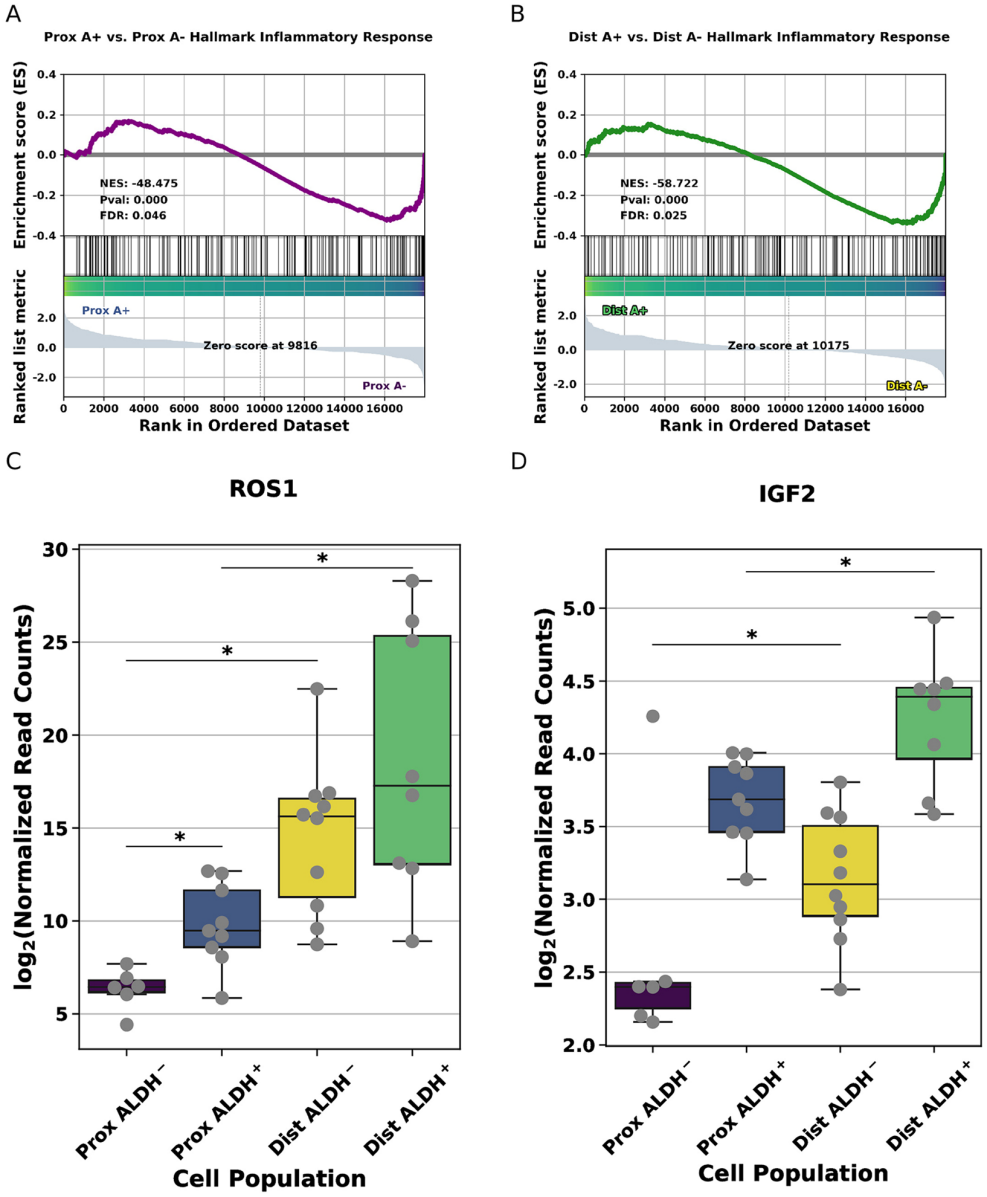
Inflammatory signaling in distal EpCAM+/ALDH− samples. (**A**) GSEA results for Hallmark Inflammatory Response in proximal EpCAM+/ALDH+ compared to proximal EpCAM+/ALDH− cell populations. (**B**) GSEA results for Hallmark Inflammatory Response in distal EpCAM+/ALDH+ compared to distal EpCAM+/ALDH− cell populations. (**C**) Boxplots displaying ROS1 expression in each of the four cell populations. * indicated FDR adjusted p-value for indicated comparison is less than 0.1. (**D**) Boxplots exhibiting IGF2 expression in each of the four cell populations. * indicated FDR adjusted p-value for indicated comparison is less than 0.1.

The same gene set enrichment analysis that identified up-regulation of Wntβ-Catenin signaling in EpCAM+/ALDH+ compared to EpCAM+/ALDH− samples also found down regulation of genes in the ‘Hallmark Inflammatory Response’ and ‘Hallmark TNFα signaling through NF-κB’ gene sets in EpCAM+/ALDH+ cells compared to EpCAM+/ALDH− cells (Supplementary Figure 8). This led us to wonder how extensively activation of inflammatory pathways might differ between different TE populations. GSEA identifies significant enrichment of the Hallmark Inflammatory Response in differentiated cell populations from both the proximal and distal TE (Fig. 5A,B). In our differential expression analysis, we noted expression changes in genes associated with malignant disease. Among these genes are *ROS1*, which is upregulated in distal EpCAM+/ALDH+ and EpCAM+/ALDH− populations compared to proximal EpCAM+/ALDH+ and EpCAM+/ALDH− populations (Fig. 5C). *ROS1* is a proto-oncogene involved in inflammatory myofibroblastic tumors^31^ and certain lung cancers^32^. We also note that IGF2 is more highly expressed in distal EpCAM+/ALDH+ and EpCAM+/ALDH− populations compared to proximal EpCAM+/ALDH+ and EpCAM+/ALDH− population (Fig. 5D). A recent study indicates IGF pathway activity along with follicular fluid in malignant transformation of TE cells^33^.

**Figure 6:**
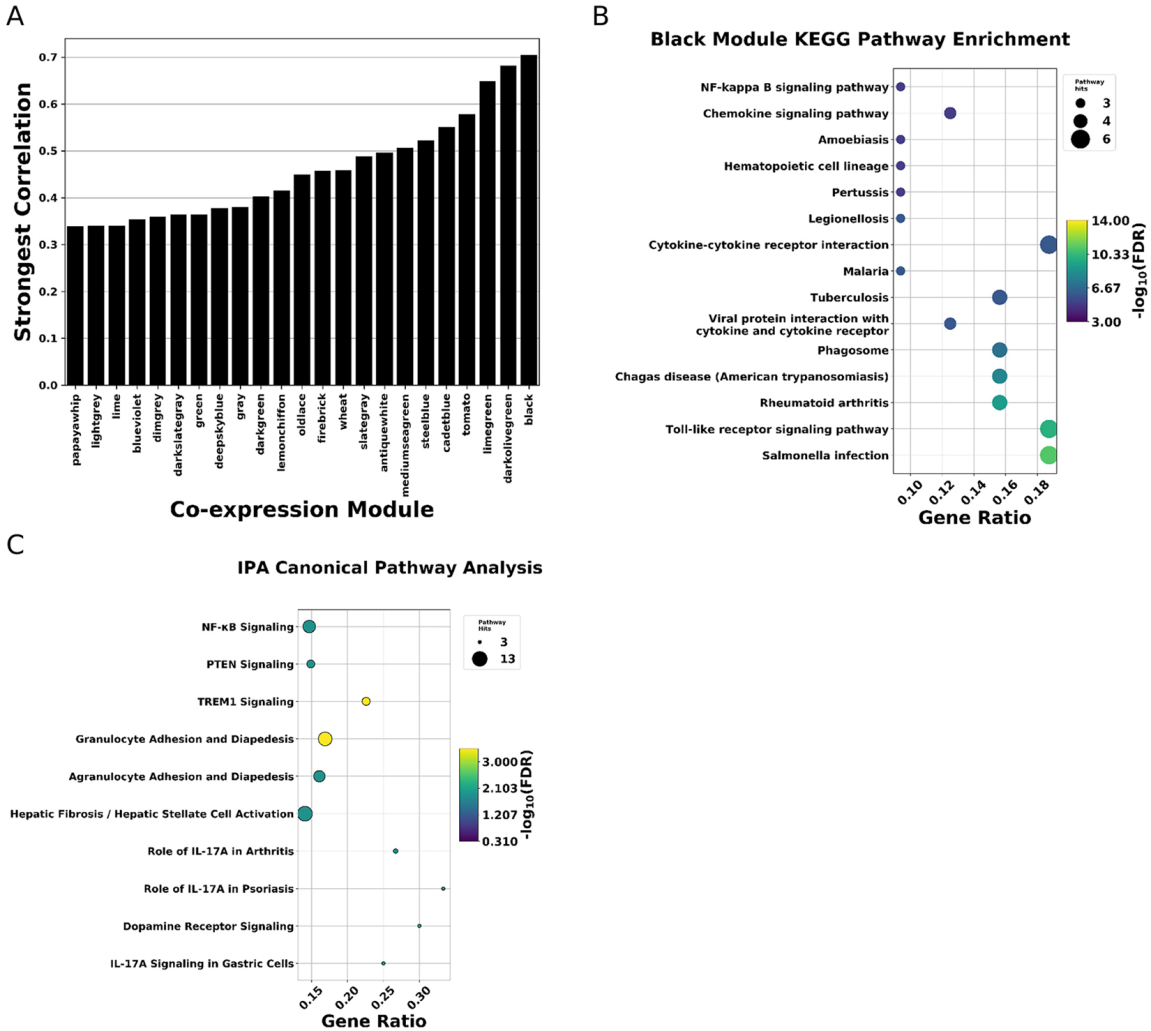
Distal TE gene co-expression networks. (**A**) Barplots indicating extent of correlation between any of the 4 cell types and a given co-expression module. Each position on the x-axis corresponds to a co-expression module. The height of the bar corresponds to the highest absolute value of correlation of that module with any of the 4 conditions. (**B**) KEGG Pathway enrichment results for ‘black’ co-expression network. (**C**) IPA heat map showing expression of pathways (names in column) in distal (left color-coded column) and proximal (right color coded column).

Intrigued by the possibility that inflammatory signaling varies across the proximal Fallopian tube regions, we decided to perform weighted gene co-expression network analysis (WGCNA^34^) to see if coordinated changes in signaling pathways could be identified between TE cell populations. We observed 22 groups of genes displaying concerted changes in expression across the 4 conditions. We found one network (‘black’) particularly interesting due to its having the strongest correlation any cell type (Fig. 6A). The genes comprising the ‘black’ module displayed a significant affinity for distal EpCAM+/ALDH− samples and a negative correlation with proximal EpCAM+/ALDH+ samples (Supplementary Figure 9). Pathway enrichment analysis indicates that the genes which comprise this co-expression network, are somewhat enriched for NF-κB signaling, as well as cytokine and toll-like receptor signaling (Fig. 6B). To corroborate findings, we decided to perform an orthogonal enrichment analysis using Qiagen’s Ingenuity Pathway Analysis Tool (IPA). Consistent with our GSEA and GO enrichment analysis NF-κB signaling was up-regulated in distal EpCAM+ samples (Fig. 6C).

### Distal Fallopian tube epithelium is enriched for gene sets characteristic of HGSC

**Figure 7:**
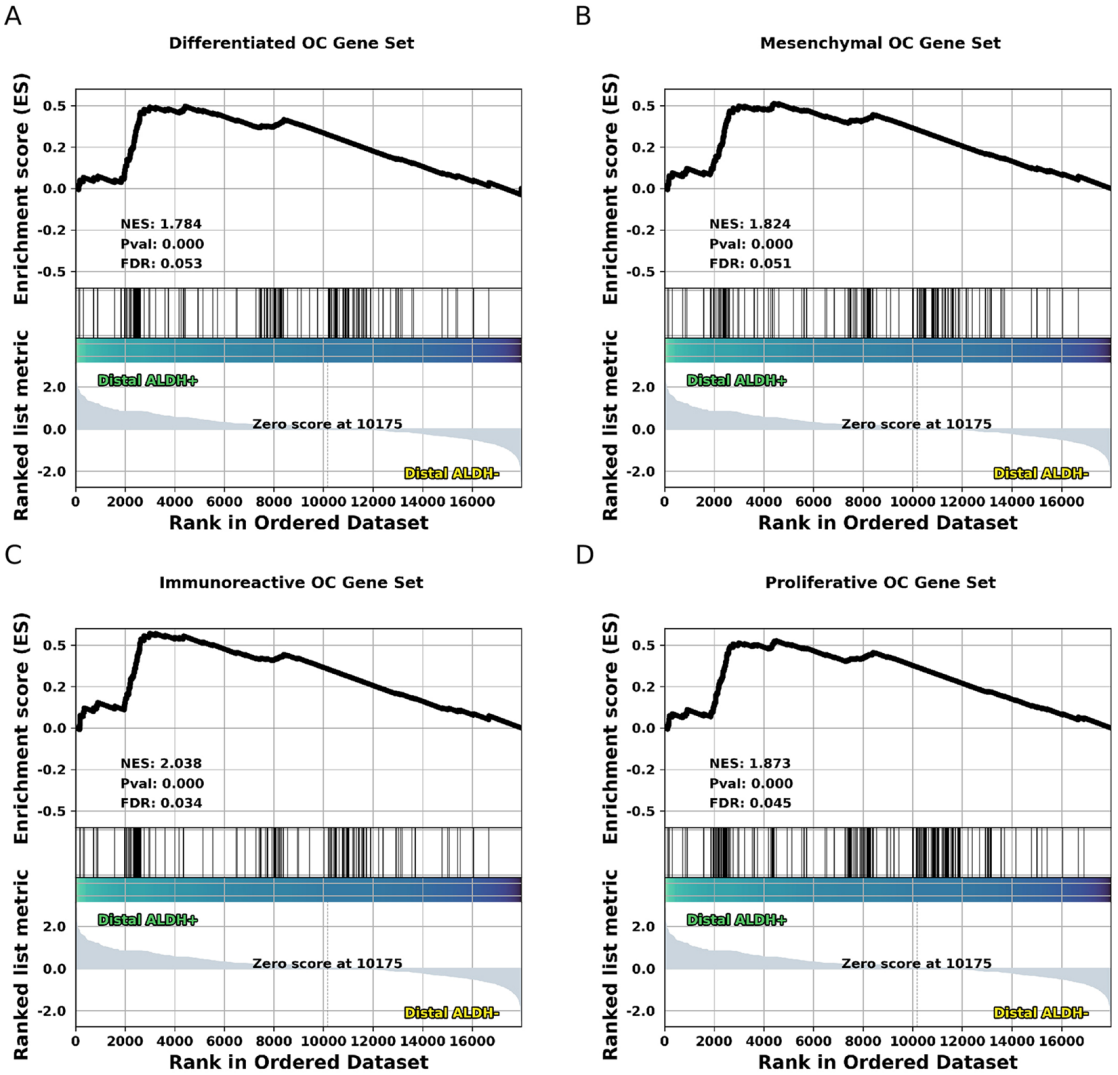
HGSC gene expression patterns in the human TE. (**A**–**D**) GSEA results for one of four gene sets corresponding to one of the 4 main molecular sub-types of HGSC identified by TCGA.

As has been mentioned, there is mounting evidence that a large fraction of HGSC originates in the distal region of the TE. HGSC encompasses at least four main molecular subtypes, but it is not clear if particular subtypes of HGSC are specifically associated with distal TE cell populations. Thus, we conducted differential expression analysis on each of the four main subtypes (1 vs. the other 3) for each molecular subtype using HGSC count data available from TCGA^35^. GSEA finds each of these four gene sets is significantly up-regulated in the distal TE (Supplementary Figure 10). Finding that the distal TE displays an upregulation of genes associated with HGSC, we wondered if distal TE ALDH+/EpCAM+ populations might express the same four gene sets more than distal TE ALDH−/EpCAM+ populations. Performing GSEA with the same four HGSC gene sets as above indicates that distal TE ALDH+/EpCAM+ populations tend to express the HGSC associated gene sets more highly, but only gene sets corresponding to the Immunoreactive and Proliferative HGSC subtypes have an FDR adjusted p-value below 0.05 (Fig. 7A–D).

## Discussion

Recent work has provided substantial insight into the relationship between HGSC and the TE^27^. However, understanding the reason for the distal TE’s susceptibility to malignant transformation remains challenging, and information about the proximal region of the TE remains sparse. Accordingly, we performed quantitative organoid and genomic studies comparing the proximal and distal regions of the TE. We observed a pronounced tendency towards organoid formation in distal compared to proximal bulk Fallopian tube patient samples. A cell population’s organoid formation tends to reflect the capacity for self-renewal and proliferation of the stem/progenitor cells within that population. Thus, differences in organoid formation between proximal and distal Fallopian tube samples are likely indicative of differences between the stem/progenitor cells of the proximal and distal regions of the Fallopian tube. Our bulk organoid formation results therefore strengthen the notion that the distal TE’s stem/progenitor cells or their environment differ in some way from those of the proximal region. These findings are consistent with observations that the distal region of the Fallopian tube more frequently contains putative HGSC precursor lesion^3,6,10^.

Isolating stem/progenitor cells from more differentiated cells is a necessary pre-requisite for understanding cell lineage dynamics in a variety of contexts. Using ALDH activity assayed by FACS/AldeRed, we have observed EpCAM+/ALDH+ populations contribute a larger fraction of a given tissue sample’s organoids than the corresponding EpCAM+/ALDH− population. This leads us to conclude that ALDH activity is a reasonable heuristic for enriching TE cell isolates for putative stem/progenitor cells.

We set out to understand how proximal and distal TE populations differ, and how these differences may help explain the evident tendency of the distal TE towards malignant transformation. Gene ontology and gene set enrichment analysis data indicate EpCAM+/ALDH+ populations (which we take to be enriched for stem/progenitor cells) employ canonical Wnt/β-Catenin signaling more extensively than cells in the (generally more differentiated) EpCAM+/ALDH− populations. Our confidence in this conclusion is strengthened by the presence of *β-Catenin* and *TCF7* among the differentially expressed genes found between putative SC/progenitor and differentiated cell enriched populations. This conclusion is consistent with observations made by the Kessler group^14^. However, our observations of primary TE gene expression data did not find significant involvement of Notch signaling, which was previously identified as a requirement for maintaining long-term TE organoid cultures. This may indicate that human TE SCs rely on other mechanisms of Wnt signaling regulation *in vivo*. However, we cannot exclude the possibility that technical limitations inherent to our study obfuscated evidence of Notch signaling.

We observe a significant enrichment of inflammatory genes in differentiated cell enriched populations from both the distal and proximal regions. IGF2 is present in follicular fluid and has recently been shown to promote malignant transformation in immortalized TE cell lines^33^. Follicular fluid is rich in inflammatory factors, and so we might expect NF-κB signaling, which frequently mediates the inflammatory response, to be upregulated in the distal TE. This expectation is fulfilled by our weighted gene co-expression network analysis and orthogonal Ingenuity Pathway Analysis, which both find increased NF-κB signaling in distal differentiated cell populations. NF-κB signaling is known to increase cellular proliferation and down-regulate P53 signaling^36,37^. Finding NF-κB signaling more active in primary human cell mRNA-seq data implicates NF-κB signaling in the distal TE’s evident susceptibility to malignant transformation and provides new, observational, evidence supporting the incessant inflammatory hypothesis. The pronounced inflammatory/NF-κB signaling Increased NF-κB signaling in differentiated cell population may lead to formation of altered niche increasing the propensity of stem cells to malignant transformation. However, we also cannot exclude that more differentiated cell population of distal TE may also to succumb to malignant transformation instead of less differentiated cell types.

The origins of HGSC are of considerable relevance to human health. We sought to assess gene expression patterns in primary human TE cell populations, to see if we might discern similarities between a particular molecular subtype of HGSC and a particular region of the TE. We find that the distal TE is significantly enriched for gene sets characteristic of HGSC subtypes. This is consistent with histological studies which find STICs occur more frequently in the distal region of the TE, though it does not help us determine for which subtype a given STIC is likely to give rise to.

While we are excited by these findings, we wish to stress some important limitations to our study. Though TNF family ligands are established regulators of NF-κB signaling, yet we do not observe significant differential expression of any TNF family genes. This may be addressed by analysis of stromally located immune cells, which may play in influencing the TE’s inflammatory response. A second peculiar finding is the absence of enrichment for cell cycle control genes. One would usually expect increased NF-κB signaling to be accompanied by a decreased DNA damage response activity and so eventual accumulation of mutations and genomic instability. We suspect our resolution is limited by the use of bulk mRNA-seq data, and the heterogeneity of epithelial cell populations in the TE. We believe that this same lack of resolution prevents us from discerning the extent to which cell number and/or cell response to genotoxic stress makes distal TE stem cell more prone to the malignant transformation. Future single cell studies will complement our current observations, garner important insight to HGSC’s pathogenesis and facilitate development of new approaches for its diagnosis, prevention and treatment.

## Materials and Methods

### Biosafety and ethical considerations

De-identified clinical samples were provided from the KU Cancer Center’s Biospecimen Repository Core Facility (BRCF) at KUMC along with relevant clinical information. Tissue specimens (Fallopian tubes - ampulla and fimbria) were obtained from women enrolled under the repository’s IRB approved protocol (HSC #5929) and following U.S. Common Rule. All patients provided written, informed consent in accordance with the BRCF IRB protocol. The samples were de-identified using OpenSpecimen. Samples were manipulated in a BSL II cabinet unless otherwise indicated.

### Collection and preservation of human TE samples

Proximal and distal Fallopian tube fragments were collected from standard surgical procedures for benign gynecological disease. The surgical tissue samples were collected on ice and examined by a surgical pathologist (maintaining the orientation and laterality of the sample), only anatomically normal uterine tubes were used. Excised tissue fragments of 1 to 2 cm size were immersed in ice cold sterile phosphate-buffered saline (PBS; Corning, Corning, USA, catalogue #21-040-CM), washed once and transferred into 1 ml cryopreservation medium M-TE-N1. M-TE-N1 consists of 75% TE-M1, 15% fetal bovine serum (FBS; MilliporeSigma, Massachusetts, USA, catalogue #ES009-M), and 10% dimethyl sulfoxide (DMSO; MilliporeSigma, catalogue #D2650-100ML). TE-M1 consists of 5% FBS, 4 mM L-glutamine (Corning, catalogue #25-005-CI), 1 mM sodium pyruvate (Corning, catalogue #25-000-CI), 100 units ml^−1^ 100 ug ml^−1^ penicillin/streptomycin (PS; Corning, catalogue #30-002-CI), 5 µg ml^−1^ insulin human (MilliporeSigma, catalogue #I9278),10 ng ml^−1^ epidermal growth factor human (EGF; MilliporeSigma, catalogue #E9644-.2MG), 10 ng ml^−1^ fibroblast growth factor-basic human (FGFb; MilliporeSigma, catalogue #F3133-10UG), 0.1 mM MEM non-essential amino acids (Corning, catalogue #25-025-CI), 0.1 mM MEM essential amino acids (Corning, catalogue #25-030-CI), 4% bovine serum albumin (BSA; MilliporeSigma catalogue #A3311-50G), and 500 ng ml^−1^ hydrocortisone (MilliporeSigma, catalogue #H0135-1MG) in 1:1 MCDB 105 (Cell Applications, San Diego, USA, catalogue #117-500) M199 (Corning, catalogue #10-060-CV) medium. Cryogenic vials with tissues were transferred to a minus 80 °C freezer for short-term storage and shipped on dry ice to Cornell University.

### Bulk Fallopian tube organoid culture

Human distal and proximal tubal epithelial organoid culture was based on the previously established protocol^14^ with some modifications. Briefly, each culture was created from a single Fallopian tube fragment. The snap-frozen tissues were thawed in the lab by incubating the tubes for 3–5 minutes at 37 °C in the water bath. The thawed tissues were collected in 2D media [(Advanced DMEM/F12 (ThermoFisher, Waltham, USA, catalogue # 12634-028), supplemented with 5% FBS, 4 mM L-GlutaMax-I (ThermoFisher, catalogue #35050061), 10 µM Rho Kinase inhibitor Y-27632 (ROCKi; MilliporeSigma, catalogue #688000), 12 mM HEPES (ThermoFisher, catalogue # 15630-080), 10 µg ml^−1^ EGF human, 100 units ml^−1^ 100 ug ml^−1^ PS] and incubated for 3 minutes. The tissues were washed at least three times with PBS to remove extra blood and debris. The tissues were dissected into 2-mm sections and then minced finally using surgical blades. The tissue homogenate was incubated in 10 ml Digestion Buffer containing 0.5 mg ml^−1^ collagenase type I (ThermoFisher, catalogue #17100-017) in Advanced DMEM/F12, medium containing 12 mM HEPES and 3 mM CaCl_2_ for 45 min at 37 °C in a water bath with rigorous shaking every 10 minutes. The tissues were minced again, centrifuged at 300xg at 22 °C, pellets were suspended in 30 ml 2D media, passed through a 100, 70, and 40 µm cell strainer. The collected cell suspension was then spun at 300xg for 7 minutes at +4 °C and the cell pellet was suspended in 3D media. The 3D media consisted of Advanced DMEM/F12, 10% R-Spondin1 conditioned medium, 25% Wnt3a conditioned medium, 4 mM L-GlutaMax-I, 12 mM HEPES, 1% N2 (ThermoFisher, catalogue # 17502048), 2% B27 (ThermoFisher, catalogue # 12587010), 100 ng ml^−1^ FGF10 human (Peprotech, Rocky Hill, USA, catalogue # 100-26-25ug), 1 mM Nicotinamide (MilliporeSigma, catalogue # N0636-100G), 10 µM ROCKi, 100 ng ml^−1^ Noggin human (Peprotech, catalogue #120-10C-20ug), 2.5 µM transforming growth factor-β Receptor inhibitor Kinase inhibitor VI (TGF-β Ri Ki VI; MilliporeSigma, catalogue #616464-5MG), 10 ng ml^−1^ EGF human, 1 µg ml^−1^ Osteopontin (OPN; MilliporeSigma, catalogue #120-35), 100 units ml^−1^ 100 ug ml^−1^ PS.

A total of 5 × 10^4^ cells were suspended in 3D media and mixed with growth factor reduced Phenol Red free Matrigel (Corning, catalogue #356231) in the ration of 30:70. This mixture was gently spread around the rim of a 12 well plate (rim assay). The plates were allowed to incubate for 20 minutes at 37 °C in a 5% CO_2_ incubator. Once the Matrigel was solidified, 500 µl of 3D media was added to rim assays and incubated at 37 °C. 10 µM p38 inhibitor (p38i; MilliporeSigma, catalogue # S7067-5MG) was added to the 3D media for the first 4 days and discontinued thereafter. The media was changed every second day and depending on the culture density the rim assay was passaged every 12 days.

Centrifugation was carried out at 4 °C and 300xg unless otherwise noted. Statistical analysis on organoid data was performed with unpaired student *t*-test. All data represented in the figures with mean ± SD. A difference was considered statistically significant at a value of *P* < 0.05.

### Immunofluorescence analysis of organoids

Immunofluorescence analysis of paraformaldehyde-fixed paraffin embedded or frozen organoids was carried out using modified previously established^38,39^. Briefly, at 22 °C the culture medium from individual organoid rim assays was removed without disturbing the organoid/Matrigel rim mixture. The assay plate was placed on ice and 1 ml of ice cold Fixation buffer was added for 3.5 hours. Fixation buffer consists of 4% paraformaldehyde in 1x PME buffer. A 10x PME buffer consists of 500 mM 1, 4-Piperazinediethanesulfonic acid (PIPES; Bioworld, Dublin, USA, catalogue #41620140-1), 25 mM Magnesium Chloride, and 0.5 M Ethylenediaminetetraacetic acid (EDTA; MilliporeSigma, catalogue #AM9260G). After the fixation, continue the work at 22 °C. The Fixation buffer was taken out from the middle of the wells, followed by addition of 0.5 ml PBS supplemented with 0.2% Triton X 100 (MilliporeSigma, catalogue #T8787-50ML and 0.05% Tween (MilliporeSigma, catalogue #T2700-100ML). The organoid suspension was collected in a 1.7 ml centrifuge tube. Wide bore yellow tips were used from this point. Organoids were centrifuged at 300xg at 4 °C, washed three times with PBS 0.2% Triton X 100 0.05% Tween, and once with PBS. The organoid pellet was suspended for dehydration in 600 µl 70% ethanol and incubated overnight at 22 °C. The next day the organoid pellet was dissolved (with taking out as much 70% ethanol as possible) in 50 µl of melted Histogel (ThermoFisher, catalogue #17985-50). The suspension forming a droplet was pipetted on a Parafilm lined petri dish and solidified at 4 °C for 10 minutes. The solidified Histogel droplet containing organoids was stored in 70% ethanol and later processed for paraffin embedding. The organoids were sectioned 4 µm thick and subjected to immunofluorescence staining using xylene deparaffinization and serial rehydration over a graded ethanol series. The antigen retrieval was performed using 10 mM sodium citrate buffer at pH 6.0 for 10 minutes. The primary antibodies against PAX8 (Abcam, Cambridge, UK, catalogue #ab189249), ALDH1A1 (Abcam, Cambridge, UK, catalogue #ab52492), and ACTUB (Sigma, St. Louis, USA, catalogue #T6793) were incubated in a humidified chamber overnight, followed by incubation with secondary antibodies (Donkey anti-Rabbit IgG (H + L) and Donkey anti-Mouse IgG (H + L) Alexa Fluor 488) for 1 hour at room temperature. Sections with no primary antibody served as negative controls. The stained sections were mounted in ProLong Diamond Antifade Mountant with DAPI reagent (Thermo Fisher). Confocal images were acquired using a Zeiss LSM 710 confocal microscope through the Cornell University Biotechnology Resource Center. The image data was merged and displayed with the ZEN software (Zeiss).

### Preparation and collection of human TE FACS samples

Human Fallopian tube samples were removed from liquid nitrogen and thawed at 37 °C for 3 minutes before being removed from the cryo-preservation vial and being rinsed 3 times with 15 ml 1x PBS. Each sample was then dissected and minced to reveal as much of the mucosa as possible, any coagulated blood was scraped away. Samples were then incubated at 37 °C for 45 minutes in Digestion Buffer, shaking every 10 minutes. Samples were then collected by centrifugation, placed in 2D Culture media and mechanically dissociated using a 5 ml serological pipette. Sample fragments were then ground with a mortar and pestle using a 300 µm filter before being further dissociated with 5 strokes of a loose Wheaton Dounce homogenizer. Samples were successively filtered through 100, 70, and 40 µm mesh filters before being collected by centrifugation and being re-suspended in 2D FACS media [Advanced DMEM/F12, supplemented with 1% N2, 2% B27, 1 mM Nicotinamid, 1 mM N-Acetyl-L-Cysteine (MilliporeSigma, catalogue # A9165-25G), 10 µM ROCKi, and 100 units ml^−1^ 100 ug ml^−1^ PS]. Samples were successively filtered through 100, 70, and 40 µm mesh filters before being collected by centrifugation and being re-suspended in 2D FACS media. For detection of ALDH enzymatic activity, sample cells were suspended in the AldeRed Assay Buffer and processed for staining with the AldeRed ALDH Detection Assay (MilliporeSigma, catalogue #SCR150) according to the manufacturer’s protocol. At this point roughly 250,000 cells were set aside for the Diethylaminobenzaldehyde (DEAB, ALDH inhibitor), EpCAM, and compensation controls. DEAB control was prepared according to manufacturer’s instructions as well. Samples/isotype control were stained with EpCAM/conjugated isotype for 1 hour at 5 °C according to manufacturer’s instructions. Appropriate sample suspensions were stained with SYTOX Blue prior to sorting on a BD FACS Aria III using 450/50, 610/20, and 696/40. Sorted cells were collected directly into 750 µl Trizol-LS (Fisher Scientific) as described^40^.

### ALDH activity segregated organoid culture

As above, each mRNA-seq library was prepared from cells originating in a single Fallopian tube fragment. Approximately 3 × 10^5^ viable EpCAM+/ALDH+ and EpCAM+/ALDH− collected by FACS into FACS media (described above) after the preparation described above. Collected cells were recovered by centrifugation at 300xg for 15 min at 4 °C. Most of the remaining liquid was decanted, and roughly 50x the remaining volume in Matrigel was added to the sample and gently mixed by pipetting. 20-30 µl droplets were then plated and allowed to sit for 30–40 minutes before the addition of 250 μl T-media. Media was changed ever two days. Cultures were passaged every week. Passaging was done by dissociating the organoids by pipetting in ice-cold 3D media containing p38i. Organoid cultures were then re-plated as described above, and dissociation to single cells was verified using bright field microscopy.

### mRNA-seq library preparation and data pre-processing

As above, each mRNA-seq library was prepared from cells originating in a single Fallopian tube fragment. 3′prime mRNA-seq libraries containing unique molecular identifiers (UMIs) were prepared using Lexogen’s QuantSeq Kit (Lexogen, Vienna, Austria, catalogue # 015.24, #081.96) according to the low-input protocol. Optimal barcodes were assigned to each sample by Lexogen’s Index Balance Checker webtool (https://www.lexogen.com/support-tools/index-balance-checker/). Libraries were pooled and sequenced on an Illumina NextSeq. 500 after undergoing QC by Agilient Fragment Analyzer.

De-multiplexed FASTQ files were inspected for quality using FASTQC^41^. Reads were aligned to GRCh38 using the STAR two-pass method^42^. UMI-tools^43^ was then applied to remove duplicate reads based on their UMI. Quality score and base re-calibration were then performed according to the Genome Analysis Toolkit best practices for mRNA-seq version 3.7. Sample identity was then verified using NGSCheckMate^25^.

### Bioinformatics analysis

For single cell analysis, a read count matrix was downloaded from Gene Expression Omnibus (GSE132149) and processed using Scanpy^44^ using an approach similar to those previously described^45^. The data were batch corrected using BBKNN^46^ (trim = 50) and visualized using Uniform Manifold Approximation and Projection^47^ (Supplementary Figure 11). Louvain clustering^48–50^ (r = 1.25) was used to segregate cell clusters. SingleR^51^ and data from the Human Primary Cell Atlas^52^ were used to identify the cell types corresponding to those clusters. Deconvolution of bulk mRNA-seq samples was performed using BSEQsc^26^ and quasi-likelihood F-tests to determine T-cell or smooth muscle contamination accounted for a statistically significant amount of variation in any gene’s expression were implemented in edgeR^53^.

For bulk mRNA-seq, a raw read count matrix was generated using the featureCounts function of the Rsubread R package^54^. Background and technical noise were reduced using the RUV-seq R package^55^ before differential expression analysis. Read count normalization and gene differential expression calls were made with DESeq. 2^29^. Gene and Disease Ontology enrichment analysis was carried out using the clusterProfiler and DOSE R packages^30,56^. Gene Set Enrichment Analysis (GSEA) was performed using the GSEApy python package^28,57^. Weighted Gene Co-expression Network Analysis (WGCNA) was performed using the Weighted Gene Co-expression Network Analysis R package^34,58^. Ingenuity pathway analysis was done using the 1,000 most divergently expressed genes between all proximal EpCAM+ samples and all distal EpCAM+ samples using ‘epithelial pathways’ as a background set. For TCGA OV Analysis Gene set’s typifying the four main molecular sub-types of HGSC were obtained from TCGA array data^59^ and supplemented using TCGA mRNA-seq data obtained using the TCGAbiolinks R package^60^. Additional information is available on GitHub (https://github.com/imr923/prox_vs_dist_TE_paper). Raw data associated with our bulk mRNA-seq samples is available through the Gene Expression Omnibus (https://www.ncbi.nlm.nih.gov/geo/), accession number GSE150283.

For enrichment analysis of follicular fluid proteins, data from a prior publication^17^ was retrieved from ProteomeXChange (http://www.proteomexchange.org/, data set PXD008073) and conducted as described above.

## Supplementary information

Supplementary Figures.

Supplementary Table 1.

Supplementary Table 2.

Supplementary Table 3.

Supplementary Table 4.

Supplementary Table 5.

Supplementary Table 6.

Supplementary Table 7.

Supplementary Table 8.

Supplementary Table 9.

Supplementary Table 10.
