# Supplementary material for: WNT and inflammatory signaling distinguish human Fallopian tube epithelial cell populations
Source: Sci Rep. 2020 Jun 17;10:9837. doi: 10.1038/s41598-020-66556-y (PMC7300082; doi:10.1038/s41598-020-66556-y)
Supplement: Supplementary file 1 — Supplementary Figures. [file 41598_2020_66556_MOESM1_ESM.pdf]

Supplementary Information

WNT and inflammatory signaling distinguish human Fallopian tube epithelial cell populations

Ian M. Rose[1], Mallikarjun Bidarimath[1], Alex Webster[2], Andrew K. Godwin[2, 3], Andrea Flesken-Nikitin[1, 4] and Alexander Yu. Nikitin[1, 4]

[1]Department of Biomedical Sciences and Cornell Stem Cell Program, Cornell University, Ithaca, New York, USA; [2]University of Kansas Cancer Center, Kansas City, Kansas, USA, [3]Department of Pathology & Laboratory Medicine, University of Kansas Medical Center, Kansas City, Kansas, USA

A

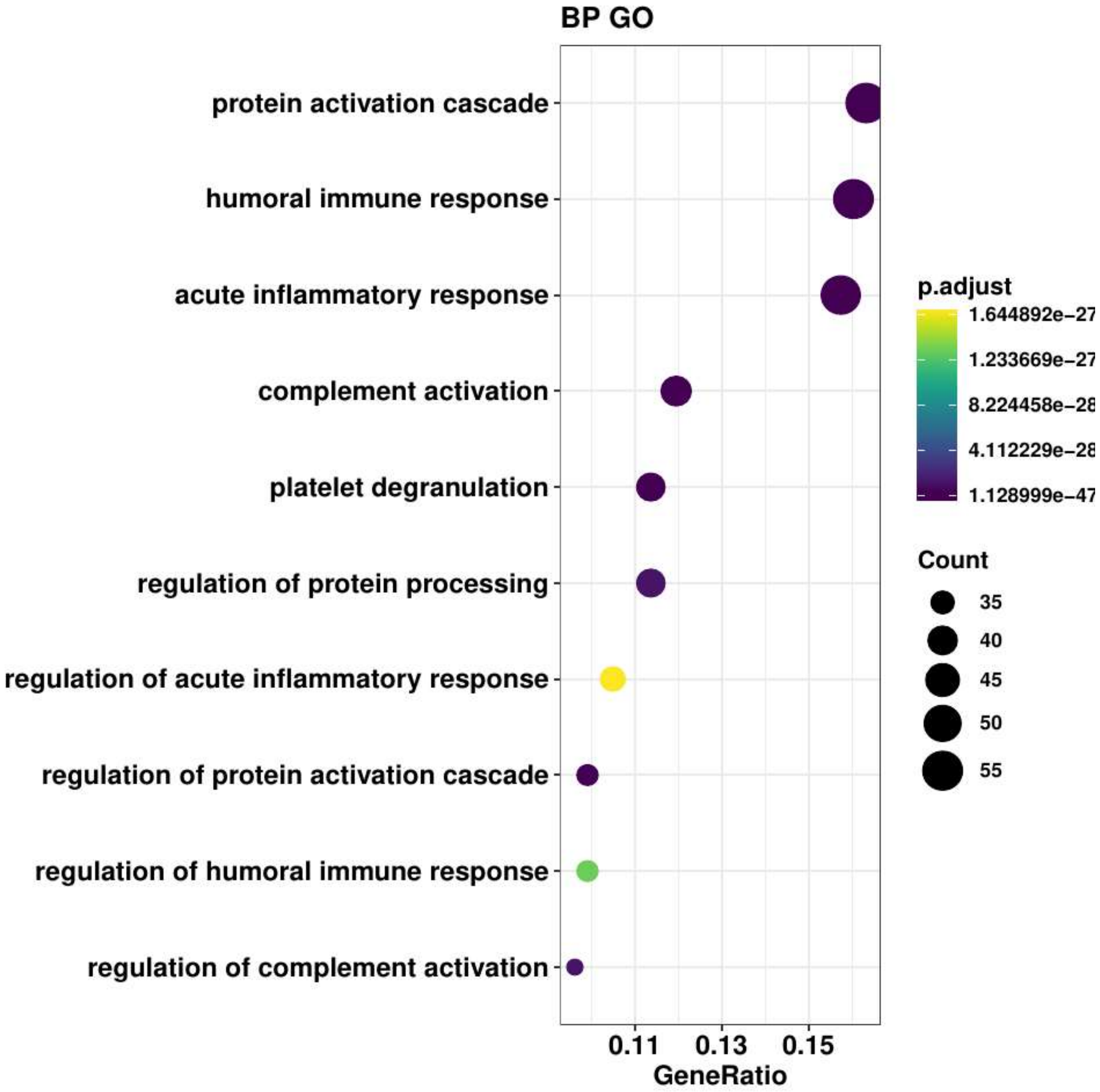

Supplementary Figure 1. Follicular fluid protein ontology enrichment. Dotplot indicating enriched GO biological process terms for proteins found in follicular fluid mass spectroscopy data (Lewandowska *et al.* 2019).

A

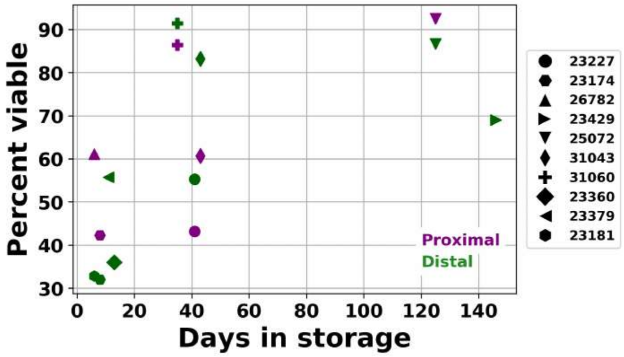

B

	sum_sq	df	F	PR(>F)
C(region)	77.53136363636361	1.0	4.652524712505728	0.05639713610633472
C(individual)	897.2418939393947	11.0	4.8947230559462485	0.009113084567949192
Residual	166.64363636363632	10.0	nan	nan

C

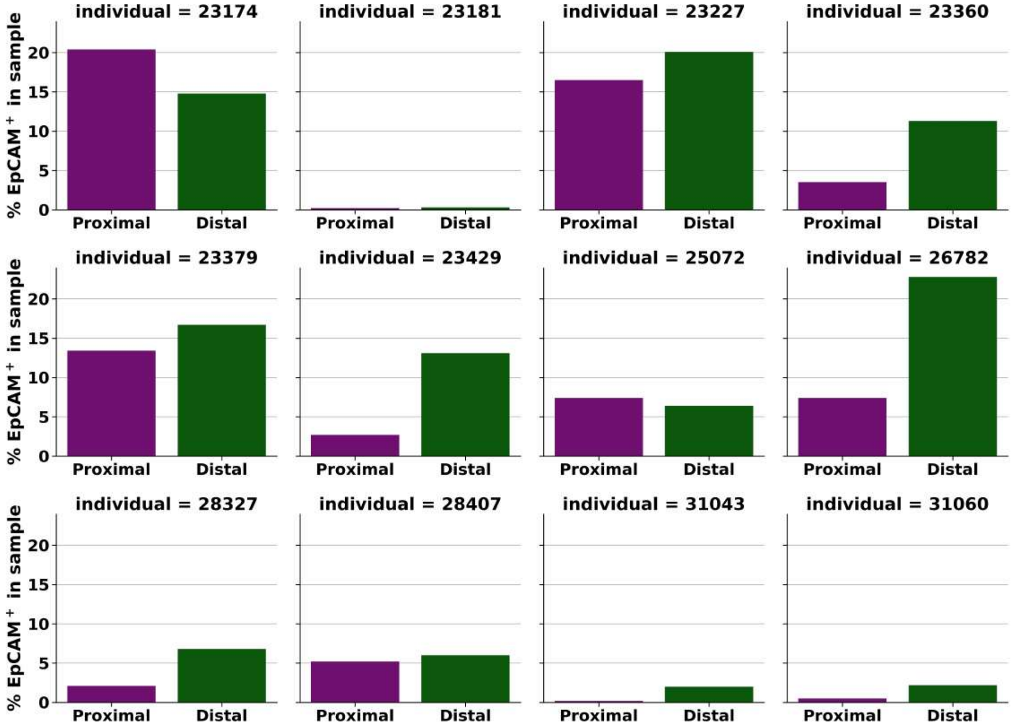

Supplementary Figure 2. Effect of storage durations and Fallopian tube fragment region. (A) Scatter plot displaying the time each sample was in storage (x-axis) against the total percentage of viable cells in that sample according to SYTOX blue staining in FACS experiments. Purple and green markers correspond to proximal and distal regions respectively. Marker shape corresponds to the individual that donated each Fallopian tube fragment. (B) ANOVA results for the effect of individual and region on number of EpCAM+ cells observed in FACS assays. (C) Bar plots displaying percentages of EpCAM+ cells in proximal and distal Fallopian tube fragments.

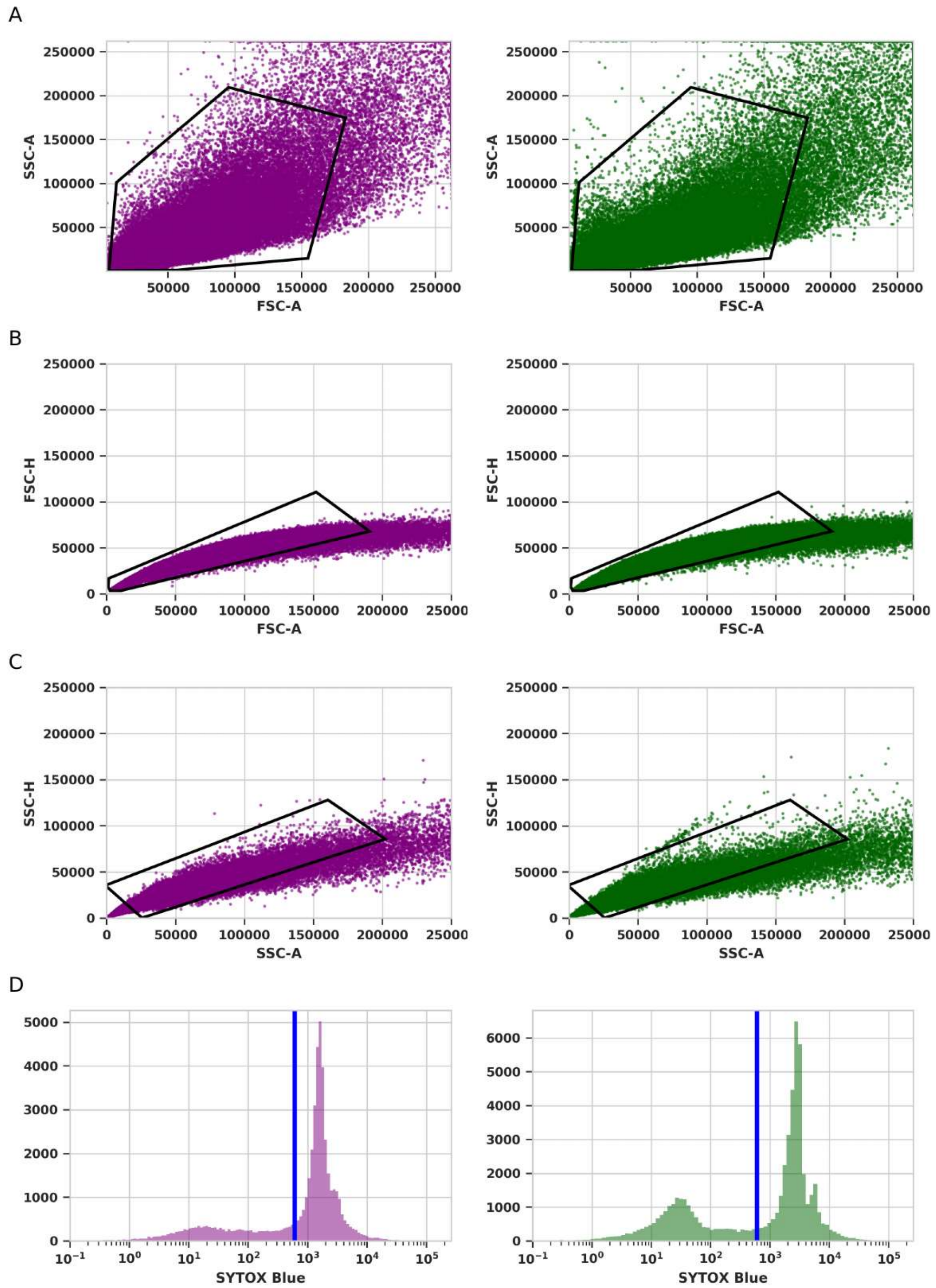

Supplementary Figure 3. Representative FACS light scatter and viability dye gating. (A) Forward vs. side scatter gate data for proximal (left) and distal (right). Points inside the black polygone were interrogated further. (B) Forward scatter area vs. forward scatter height gate data for proximal (left) and distal (right). Points inside the black polygone were interrogated further. (C) Side scatter are vs. side scatter height gate data for proximal (left) and distal (right). Points inside the black polygone were interrogated further. (D) Histograms indicating staining for the viability dye SYTOX Blue. The blue line on both the proximal (left) and distal (right) plots corresponds to the threshold, below which, a cell was deemed viable. Cells that fell to the right of the threshold were discarded.

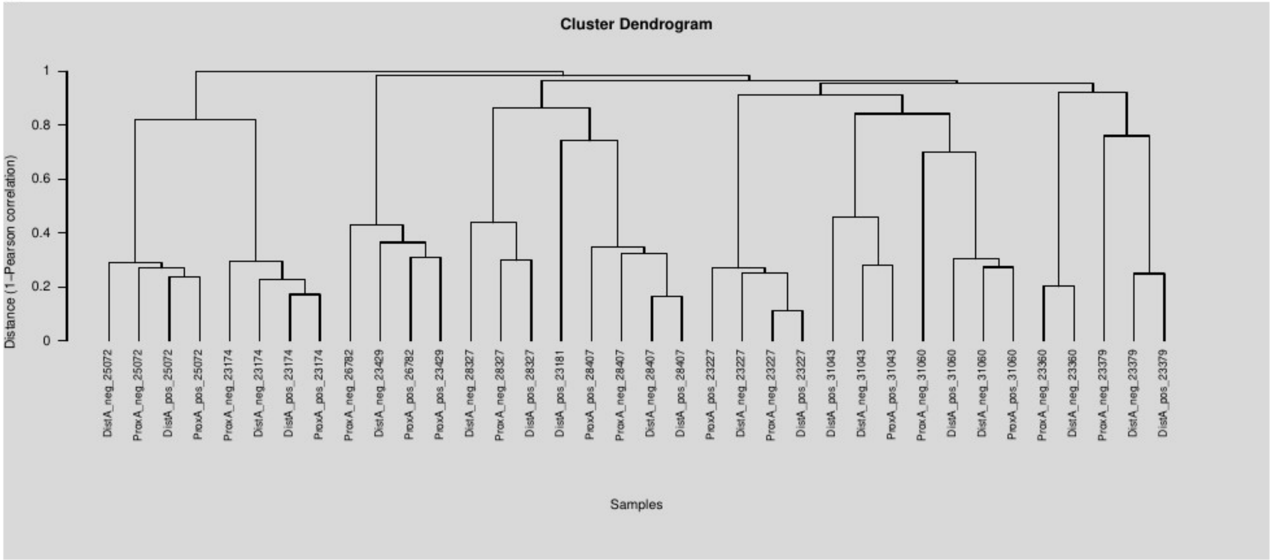

Supplementary Figure 4. NGS Checkmate dendrogram. SNP correlation matrix is used to cluster mRNA-seq samples by individual that donated material.

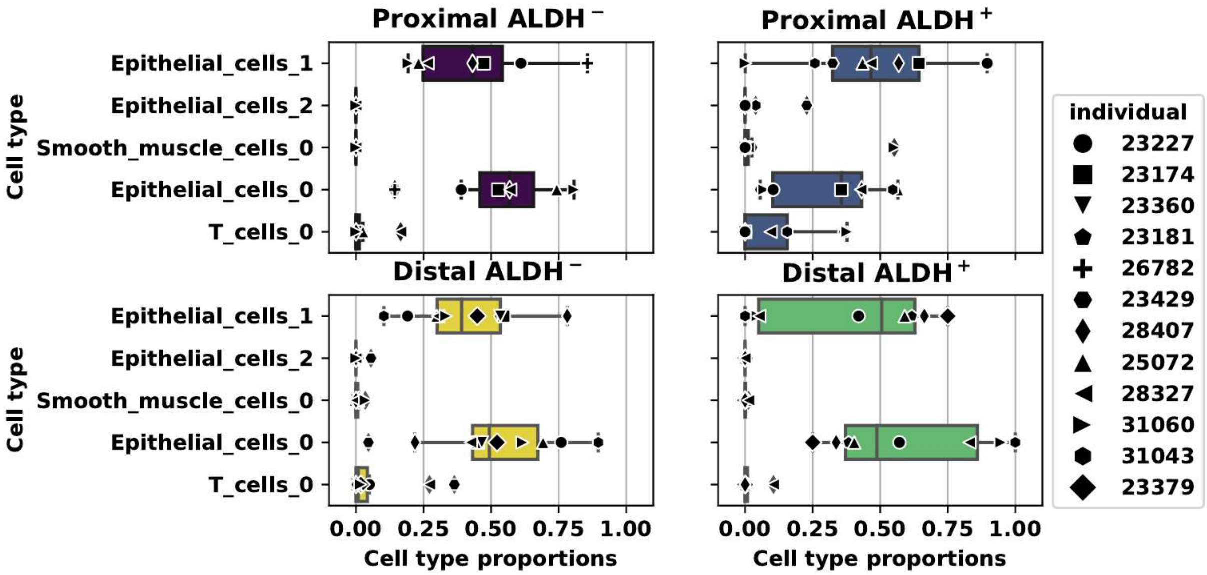

Supplementary Figure 5. Estimates of cell type composition for each mRNA-seq sample. Each of the four facets corresponds to a different TE cell population. Each shape corresponds to a different mRNA-seq sample, the specific shape indicates which individual that mRNA-seq sample originated from. Each row on the y-axis corresponds to a different cell type. The x-axis indicates what fraction of the sample is made up by the cell type indicated on the y-axis.

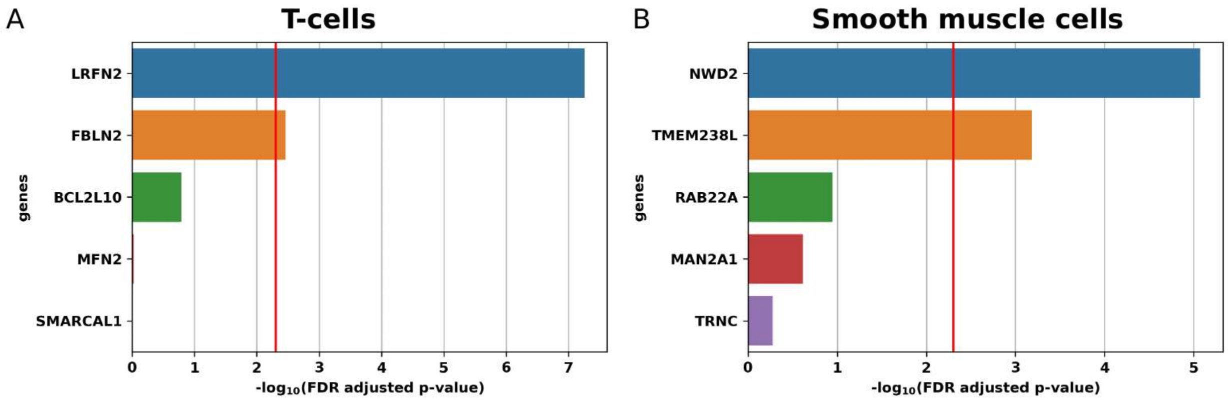

Supplementary Figure 6. Genes with a significant variation associated with contaminating cell type. (A) Bar plots indicating which genes have a significant portion of their variance explained by the contaminating T-cells. The red line corresponds to a FDR adjusted p-value of 0.1. Genes with bars that reach, or extend to the right of the red line, have a significant amount of their variance explained by contaminating T-cells. (B) Bar plots indicating which genes have a significant portion of their variance explained by the contaminating smooth muscle cells. The red line corresponds to a FDR adjusted p-value of 0.1. Genes with bars that reach, or extend to the right of the red line, have a significant amount of their variance explained by contaminating smooth muscle cells.

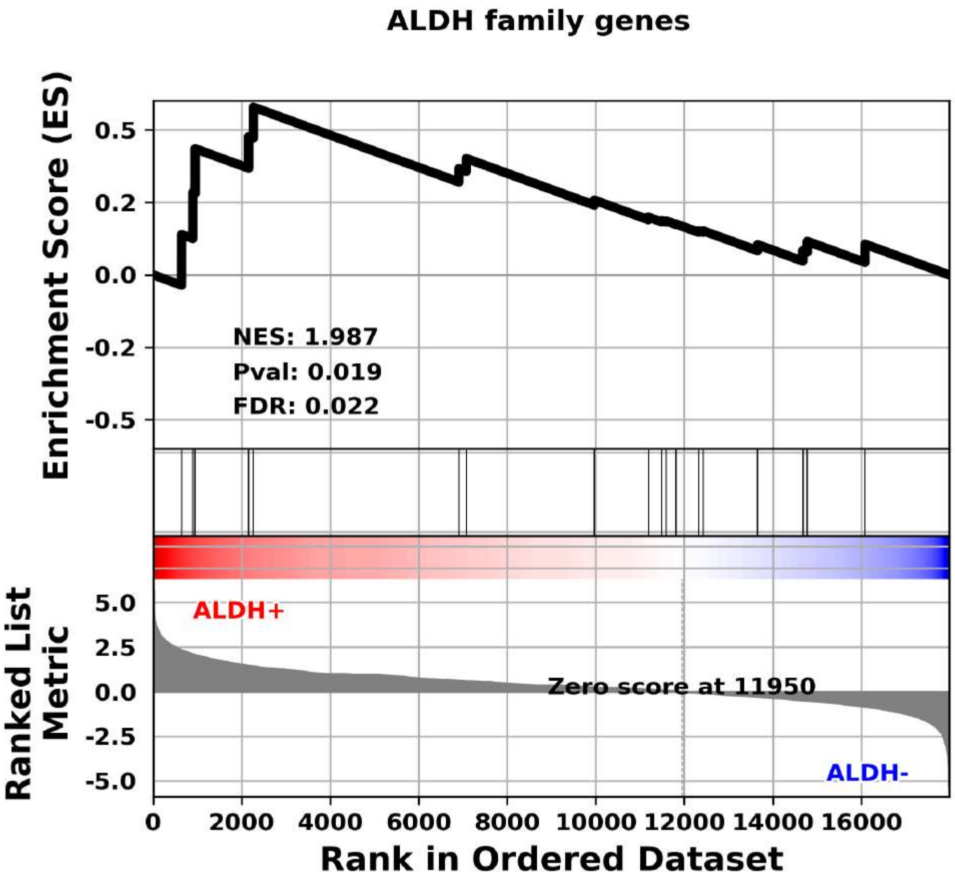

Supplementary Figure 7. ALDH family GSEA. GSEA using all ALDH family genes, which are present in primary human TE mRNA-seq data as a gene set.

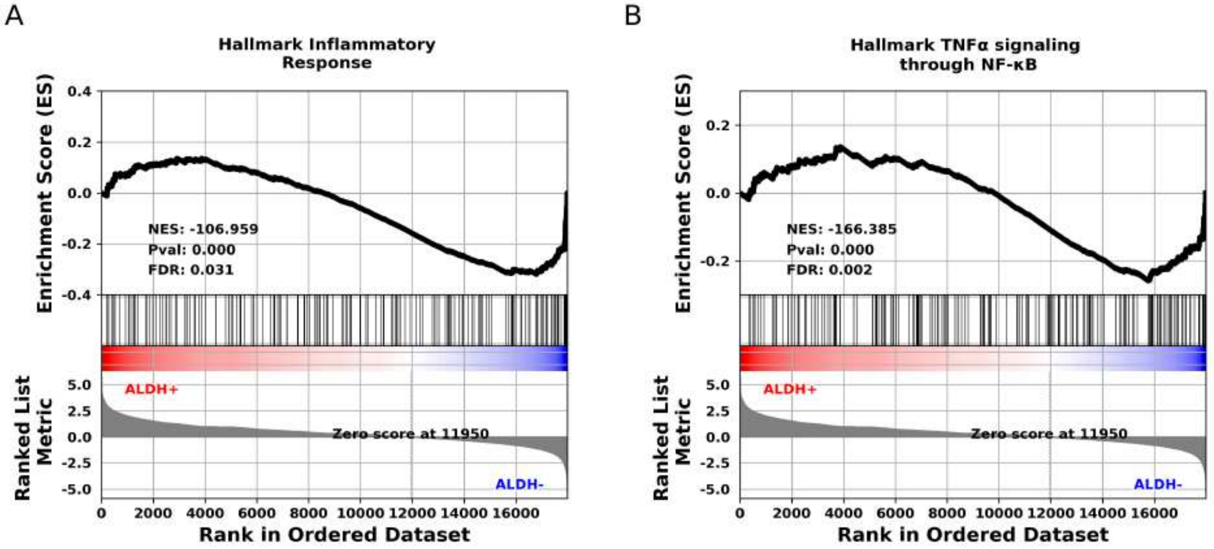

Supplementary Figure 8. Two most statistically significant Hallmark gene set enrichment results. (A) GSEA plot showing results for Hallmark Inflammatory Response gene set in all EpCAM+/ALDH+ samples compared to all EpCAM+/ALDH- samples. (B) GSEA plot showing results for Hallmark TNFα signaling through NF-κB gene set in all EpCAM+/ALDH+ samples compared to all EpCAM+/ALDH- samples.

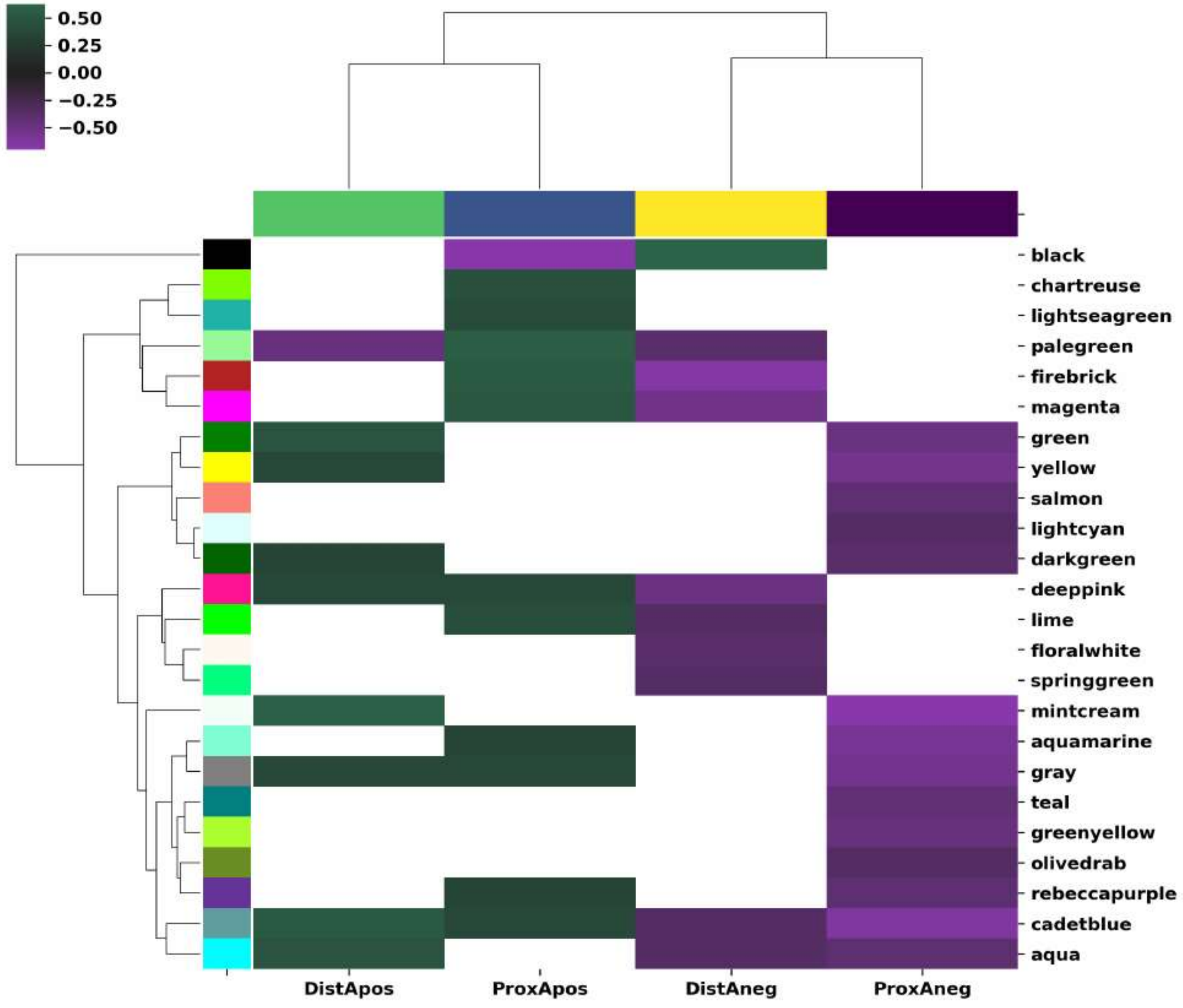

Supplementary Figure 9. Weighted Gene Co-Expression Network analysis. Each row corresponds to a gene expression module identified using WGCNA. Each column corresponds to one of the four cell types. Every colored (non-white) cell corresponds to a statistically significant ($p<0.05$) association between a co-expression module and a cell type. Green cells indicate a positive association between the co-expression module and the cell type. Purple cells indicate a negative correlation between the co-expression module and the cell type.

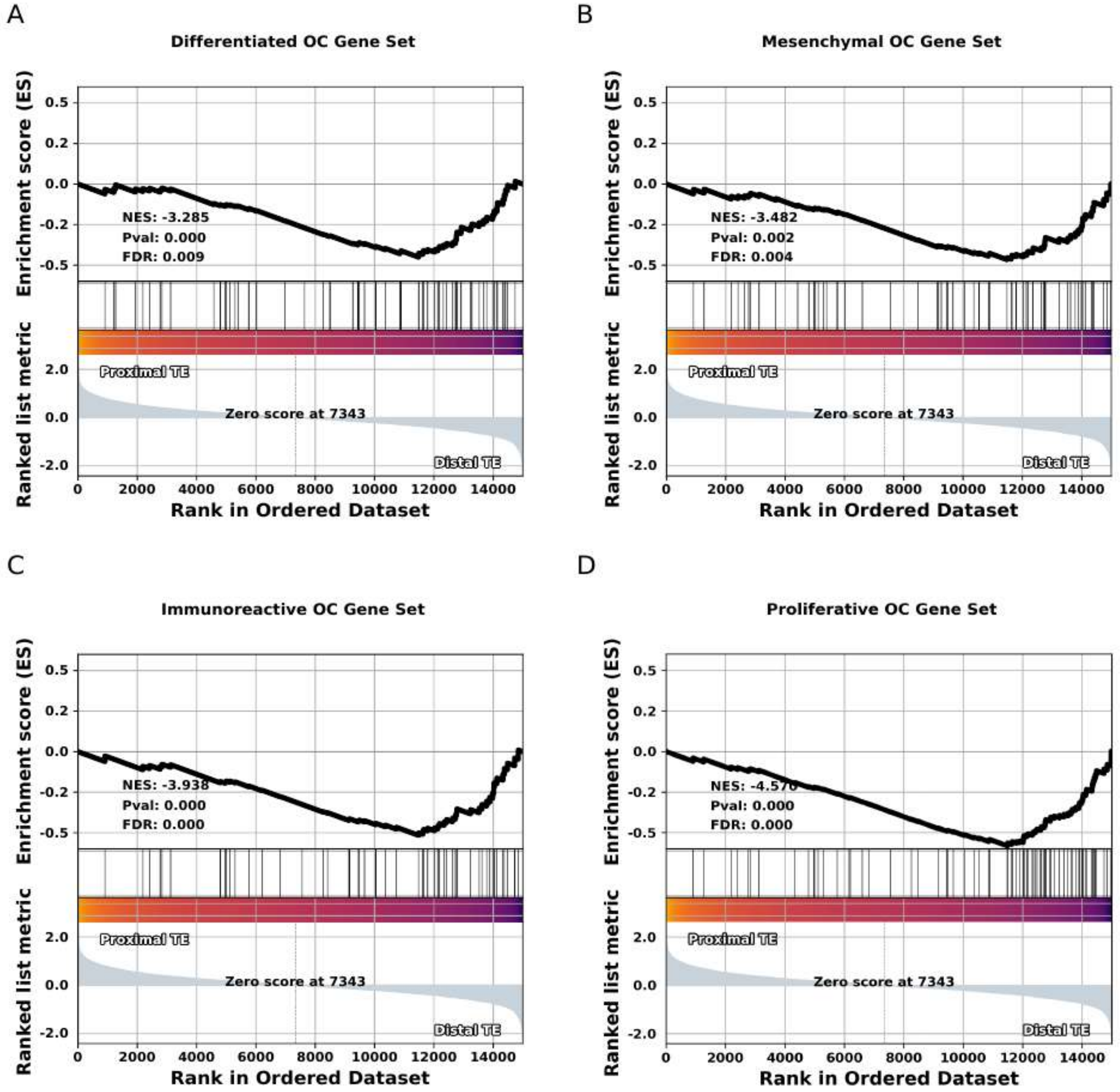

Supplementary Figure 10. HGSC gene expression patterns in the proximal vs distal human TE. (A-D) GSEA results for one of four gene sets corresponding to one of the 4 main molecular sub-types of HGSC identified by TCGA. All enrichment results shown pertain to all proximal region samples compared to all distal region samples.

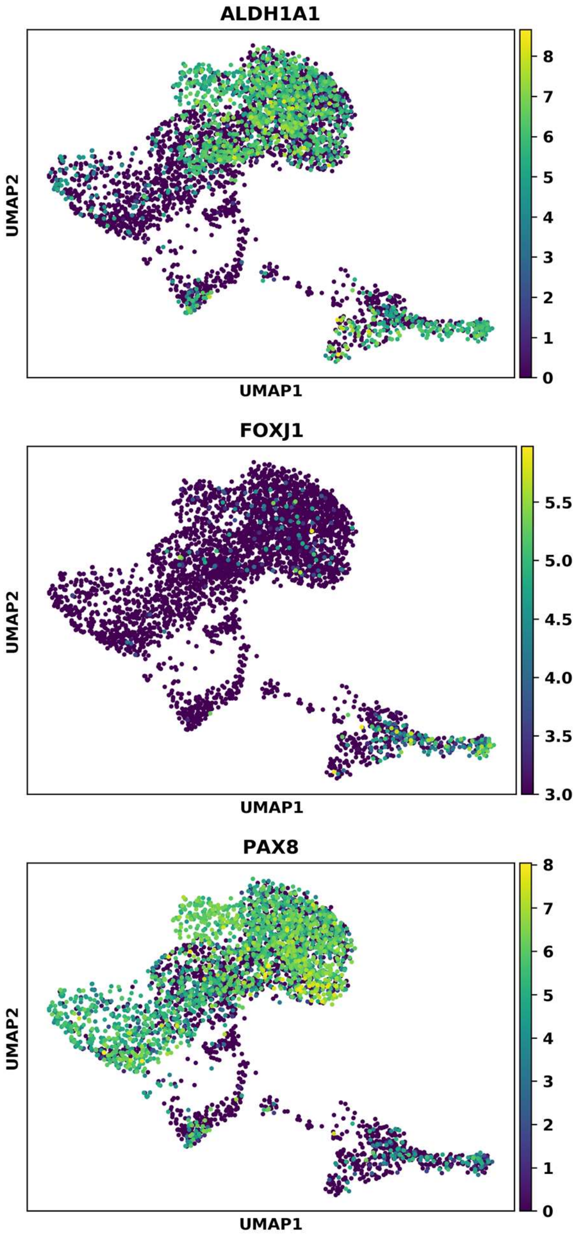

Supplementary Figure 11. Expression of ALDH1A1, PAX8 and FOXJ1 in single-cell mRNA-seq published by Hu et al. 2020. Each UMAP plot corresponds to the gene indicated above that plot. Each point on each UMAP corresponds to an individual cell. The color of each cell corresponds to the log2(normalized read counts) observed in that cell. At a glance, there does not seem to be an obvious association in expression between ALDH1A1, PAX8 and FOXJ1.
